# Emphasis on Financial vs Nonfinancial Criteria in Employer Benefits’ Measurements

**DOI:** 10.1001/jamahealthforum.2024.5229

**Published:** 2025-01-31

**Authors:** Jeffrey Pfeffer, Esther Olsen, Sara J. Singer

**Affiliations:** 1 Graduate School of Business, Stanford University, Stanford, California; 2 School of Medicine, Stanford University, Stanford, California; 3 School of Medicine, Graduate School of Business, Stanford University, Stanford, California

## Abstract

**Importance:**

Few studies have examined the extent to which employers emphasize financial over nonfinancial criteria in measurement, reporting, and decision-making about health care benefits.

**Objective:**

To measure and identify factors associated with financial over nonfinancial emphasis in employer decision-making about health benefits.

**Design, Setting, and Participants:**

A survey was administered to a nationally representative sample of US employers to assess the extent of employers’ emphasis on benefits plans’ costs over member experience, access to care, and equity, and on financial vs other considerations when choosing third-party benefits administrators. The sample included in-company human resources administrators from randomly selected nongovernmental organizations with at least 50 employees. The survey was administered in 2 waves: May 2022 to July 2022 and November 2022 to April 2023.

**Exposure:**

The survey included 41 multipart questions capturing information about the respondent, company, company interactions with benefits consulting firms and benefits administrators, and company approach to managing employee health benefits.

**Main Outcomes and Measures:**

Main outcomes were proportion of financially oriented measures that internal benefits administrators and external benefits consultants use and importance of financial vs other factors in companies’ choice of third-party administrators.

**Results:**

Of 1159 companies sampled, 251 (22%) responded; 30 with less than 50 employees were excluded. Of the 221 remaining companies, 147 (67%) used a benefits consulting firm. The companies and their benefits consultants focused on financial over nonfinancial performance dimensions in decision-making. While 125 companies (74%) tracked trends in health benefits costs and 109 (64%) tracked spending on the highest cost cases, only 14 (8%) tracked time employees spent having questions answered, and 12 (7%) tracked how often employees delayed receiving care because of an insurance company’s actions. This financial focus was largely independent of organizational characteristics and other potential explanatory factors. Of 37 paired differences comparisons in the proportion of financial vs nonfinancial items, only 6 proportions (16%) differed significantly, with differences in proportions of 0.22 or less.

**Conclusions and Relevance:**

In this survey study, US employers emphasized financial over nonfinancial criteria in their measurement and decision-making about health benefits. To improve health plan performance, employer measurement and decision-making must emphasize both nonfinancial and financial criteria.

## Key Points

**Question:**

Do employers emphasize financial vs nonfinancial measures in decision-making about employee health care benefits, and if so, what factors are associated with financial emphasis?

**Findings:**

In a representative sample of 221 respondents involved in employer health benefits oversight, employers and their health benefits consultants focus more on financial than nonfinancial performance dimensions in both decision-making and measurement. Financial emphasis was associated with using a benefits consulting firm and with not seeking employee feedback about benefits.

**Meaning:**

To improve broader aspects of health plan performance, employer measurement and decision-making must emphasize nonfinancial as well as financial criteria.

## Teaser

This survey study evaluates how much emphasis is placed on financial vs nonfinancial factors in employer decision-making about health care benefits.

## Introduction

Measurement matters because it focuses management attention on what is being measured.^1^ Indeed, the importance of measurement in health care settings has led to a proliferation of measures and a concomitant call to focus measurement on only the most important and impactful indicators.^2^

According to KFF, “60.4% of people [in the US] under age 65, or about 164.7 million people, had employment-sponsored health insurance in 2023.”^3^ Given employers’ large role in the health care ecosystem, what employers measure may affect access to care and how employees and their families interact with health care professionals.

Previous research studying employers’ role in employee health demonstrated, in a small sample of purposely sampled progressive companies, an enormous concern with costs and changes in costs.^4^ We wondered if this experience was typical because if it was, the overemphasis on the financial and cost aspects of health benefits and, the possible neglect of other health plan dimensions, following the measurement-performance connection, could negatively affect health care outcomes.

The present study sought to examine whether purchasers of employee health benefits emphasize financial considerations in their measurement, reporting, and decision-making about health benefits and benefits administration. We argue that in a market-driven or quasi–market-driven system, what purchasers measure and prioritize in their decisions about benefits will influence what their vendors emphasize. Simply put, as the quality movement and other management literature have long found, measurement matters for how organizations operate and what people within the organizations prioritize and do.^5^

We note that few studies have examined the measures and criteria used in decision-making by the employers that purchase and provide health benefits. We use new survey data to assess the extent to which US employers emphasize financial over nonfinancial criteria in decision-making about health care using these indicators: (1) proportion of measures that internal benefits administrators and external benefits consultants use that are financially oriented; and (2) relative importance of financial vs other considerations in companies’ choice of third-party administrators.

We expected to find greater emphasis on financial compared to nonfinancial measures and criteria among employers in their oversight and decision-making about health plans. We further expected that this emphasis would be widely practiced among employers and independent of organizational characteristics and other potential explanatory factors.

## Methods

### Survey Development

For this survey study, we developed the Employee Healthcare Benefits Survey drawing on previous research^4,6^ and expert input. We followed the American Association for Public Opinion Research (AAPOR) reporting guideline. The Stanford University School of Medicine Institutional Review Board approved study procedures on an expedited basis and waived the requirement for written informed consent as participants indicated consent by completing the survey. The final survey included 41 multipart questions capturing information about the respondent and company, assessing companies’ interactions with health benefits consulting firms and health benefits administrators, and the companies’ approach to managing employee health benefits. Relevant items included consulting tasks, asked of companies that used a health benefits consultant, criteria for selecting an administrator, asked of companies that use a health benefits administrator, information included in benefits administrators’ reports, asked of companies that receive an annual report on performance of their health plan, companies’ tracking of benefits information, excluding companies that were unsure, and what companies did with any health benefits savings, excluding companies that had no savings. See the eAppendix in Supplement 1 for the survey and methodological details.

### Survey Sample

Our nationally representative sample included in-company human resources administrators of randomly selected companies drawn from Dun & Bradstreet^7^ that self-reported at least 50 employees. It excluded government-affiliated organizations and was stratified by number of employees (50-99, 100-499, or ≥500). SSRS, a professional research and survey firm,^8^ administered the survey in 2 waves: from May 2022 to July 2022 and from November 2022 to April 2023, reaching out to each organization 3 times using multiple modalities and conducting online and phone verification to identify qualified respondents. The responses for some variables reflect a subset of respondents due to the survey’s skip logic. Item missingness, unrelated to skip patterns was low, less than 5% for all items.

### Statistical Analysis

We report unweighted data to describe sample characteristics. In reporting other results, we weighted our sample to be representative of establishments in the Dun & Bradstreet database^7^ by size, geography, and industry (see the eMethods in Supplement 1 for the sample demographics summary). To investigate companies’ emphasis on financial concerns over other factors, we examined the difference in financial and nonfinancial composite measures derived from a multipart survey question or questions (see the eAppendix in Supplement 1 for the composition of composites). To identify factors associated with employer priorities about employee health benefits, we considered a set of company characteristics that we thought, before conducting the survey, might be potentially relevant, including company size, risk for health care costs, use of benefits consultant, use of benefits administrator, frequency of requesting employee feedback regarding health benefits, inclusion of employee health and well-being in company’s mission statement, having a formal plan for health and well-being, and count of company actions creating a culture of health.

We computed percentage of respondents who selected each item listed in consulting tasks (question 6), spending/user experience information in the benefits administrator report (questions 11 and 12), health information tracked by the company (question 19), and recipient of savings, if any (question 23), as well as the mean percentage across financial and nonfinancial items within each question, applying weights so that results reflect companies within the US. Similarly, we computed the mean importance score for criteria in selecting a benefits administrator (question 9), for each item individually and for the financial and nonfinancial composites, based on the weighted data. We used paired *t* tests to assess differences in financial vs nonfinancial emphasis in each series probed; we conducted tests on paired differences in proportions for question 6; questions 11 and 12; question 19; and question 23, and on the paired difference in mean importance scores for question 9. We performed weighted and unweighted multivariable linear regression modeling of the differences in proportions or means to identify factors associated with employer emphasis on financial vs nonfinancial aspects of employee health benefits, considering *P* < .05 as statistically significant with 2-sided testing. Given limited differences in results, we report weighted results.

## Results

Of 1159 companies sampled, 251 (22%) responded, and 30 that self-reported fewer than 50 employees were excluded, leaving 221 companies for data analysis. Respondents did not differ significantly from nonrespondents on available characteristics (eTable 1 in Supplement 1). A total of 119 respondents (54%) had between 100 and 999 employees; 49 (22%) had between 1000 and 9999 employees, 42 (19%) had between 50 and 99 employees, and 11 (5%) had more than 10 000 employees (Table 1).

**Table 1.** Characteristics of Companies in Analytic Sample^a^

Characteristic	Companies, No. (%) (N = 221)
Size, No. of employees	
50-99	42 (19)
100-999	119 (54)
1000-9999	49 (22)
≥10 000	11 (5)
Company insurance status	
Fully insured	120 (54)
Self-insured	92 (42)
Unsure/unknown	9 (4)
Use of a health benefits consulting firm	
Yes	147 (67)
No or unsure/unknown	74 (33)
Use of a health benefits administrator	
Yes	182 (82)
No or unsure/unknown	39 (18)
Average sick time per employee in company, d	
<3	34 (15)
3-5	76 (34)
6-10	37 (17)
>10	3 (1)
Unsure/unknown	71 (32)
Average tenure of employees in company, y	
<3	20 (9)
3 to <5	38 (17)
5 to <10	78 (35)
10 to <15	44 (20)
≥15	18 (8)
Unsure/unknown	23 (10)
Census region	
New England	7 (4)
Middle Atlantic	21 (12)
East North Central	27 (15)
West North Central	21 (12)
South Atlantic	40 (23)
East South Central	10 (6)
West South Central	19 (11)
Mountain	5 (3)
Pacific	25 (14)
Missing data	46
Industry, based on Standard Industrial Classification codes^b^	
Agriculture, forestry, and fishing	5 (2)
Construction	12 (5)
Finance, insurance, and real estate	16 (7)
Manufacturing	38 (17)
Mining	3 (1)
Retail trade	22 (10)
Services (other than health care and education)	62 (28)
Services, health care	28 (13)
Services, education	16 (7)
Transportation, communications, electric, gas, and sanitary services	8 (4)
Wholesale trade	11 (5)

^a^ Based on unweighted data.

^b^ Data source was the Dun & Bradstreet database.

### Criteria for Choosing a Health Benefits Administrator

A total of 182 companies (82%) used a health benefits administrator, such as UnitedHealth Group, Anthem, Humana, Blue Cross Blue Shield, CVS Health, or Kaiser Permanente (question 7). When choosing a health benefits administrator to manage its employees’ health benefits and the day-to-day operations of the health plan on behalf of the organization, companies favored cost criteria (question 9). Based on the weighted sample, the mean (SD) importance for cost factors, including price of administration and pricing of comparable services, was 4.5 (0.6) on a scale of 0 to 5, indicating these cost criteria were extremely important in companies’ choice of health benefits administrator. The mean (SD) importance for member experience factors, including telephone availability, ease of use of the administrator’s website, member satisfaction, quality ratings, ease of locating in-network health care practitioners, and employee health outcomes was 4.0 (0.6), with the difference from financial factors being statistically significant (paired *t* test *P* < .001) (eTable 2, question 9, in Supplement 1).

### What Companies and Their Advisors Measure and Track

**Figure 1.**
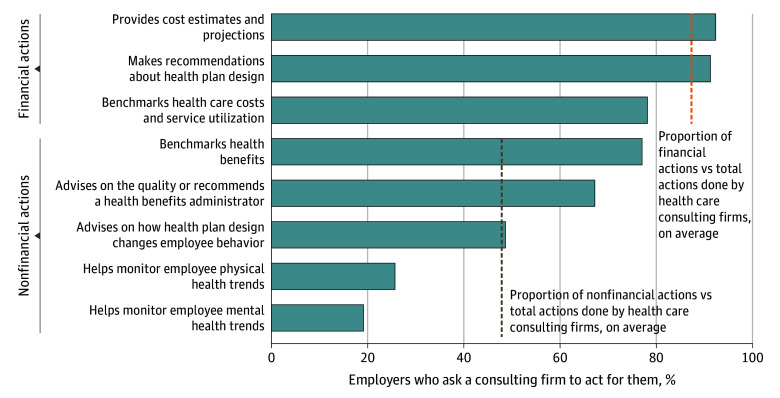
Actions Performed by Companies’ Health Benefits Consulting Firms These data were derived from the analysis of the Employee Healthcare Benefits Survey. These results are based on weighted data and are from question 6, which reads, “Which of the following, if any, does the health benefits consulting firm do for you?” Survey responses have been truncated to better fit the graph. For the full description of survey responses, see Supplement 1. Proportion of financial and nonfinancial actions vs total actions performed by the health care consulting firms was calculated as the mean for relevant actions of the mean percentage of employers that ask consulting firms to act for them, represented by dashed lines.

Overall, 147 companies (67%) used benefits consulting firms, which are often influential in companies’ decisions about the design of health plans and which benefits administrators to use (question 5). Based on weighted data, for companies that used benefits consulting firms, 132 (92%) and 131 (91%), respectively, asked them to perform tasks concerning financial actions, including providing cost estimates and making recommendations about health plan design (question 6; Figure 1). In contrast, only 37 (26%) and 27 (19%) asked the benefits consulting firms to monitor employee physical or mental health trends, respectively. The mean (SD) number of financial actions that companies asked the consulting firm to perform per respondent was 2.6 (0.6) (of 3 total items, 87% [8%]), and the mean number of nonfinancial actions was 2.4 (1.4) (of 5 total items, 48% [26%]). This difference in proportions was statistically significant (*P* < .001) (eTable 3, question 6, in Supplement 1).

**Figure 2.**
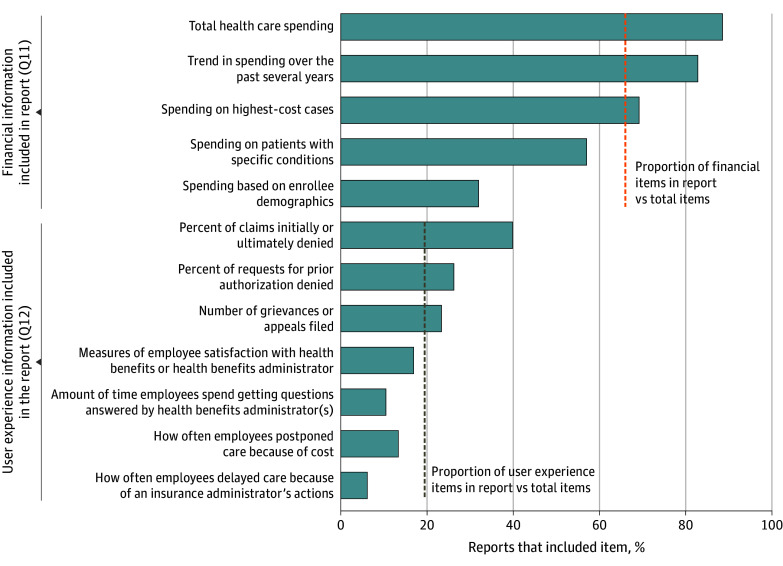
Spending and User Experience Information Included in Health Benefits Administrators’ Reports These data were derived from the analysis of the Employee Healthcare Benefits Survey. The results are based on weighted data and are from question 11 (Q11) and question 12 (Q12), which read, “Which, if any, of the following [spending/user experience] information is included in the report? Please select all that apply.” Survey responses have been truncated to better fit the graph. For the full description of survey responses, see Supplement 1. The proportion of financial and user experience items in the report vs total items calculated as the mean for relevant items of the percentage of reports that include the items, represented by dashed lines.

Of companies that used a benefits administrator, 106 (61%) received an annual report on the performance of their health plan from their health benefits administrator. The information these companies received emphasized health spending information more than user experience information (questions 11 and 12; Figure 2). The percentage of companies receiving reports that include each of the 5 different measures of health spending information ranged from 87 (88%) for information on total health care spending to 32 (32%) for information on spending based on enrollee demographics, such as income, gender, race, and ethnicity, for a mean (SD) of 66% (22%) of companies across the 5 measures. Excluding information on demographics, which could be considered an equity rather than a financial issue, the mean (SD) percentage across measures increases to 74% (14%) of companies.

By contrast, for the 7 categories of user experience information, the number of companies that received this information in their report ranged from 34 (40%) for claims initially or ultimately denied to only 5 (6%) for how often employees delayed filling a prescription, visiting a physician, or having a medical procedure because of an insurance administrator’s actions, such as denying authorization, for a mean (SD) of 19% (4%) of companies across the 7 user experience measures. Interestingly, although 95 companies (55%) said that member satisfaction was an extremely important criterion in their company’s choice of a health benefits administrator, only 15 companies (17%) said that they obtained information on measures of employee satisfaction with their health benefits administrator as part of their annual reports.

The mean (SD) number of financial (spending) items included in the report per respondent was 3.3 (1.5) (of 5 total items, 66% [5%]), and the mean number of nonfinancial (user experience) items per respondent was 1.4 (1.5) (of 7 total items, 19% [4%]), a significant difference in the proportion of financial vs nonfinancial items included in the benefits administrator’s annual report (paired *t* test *P* < .001) (eTable 4, questions 11 and 12, in Supplement 1).

**Figure 3.**
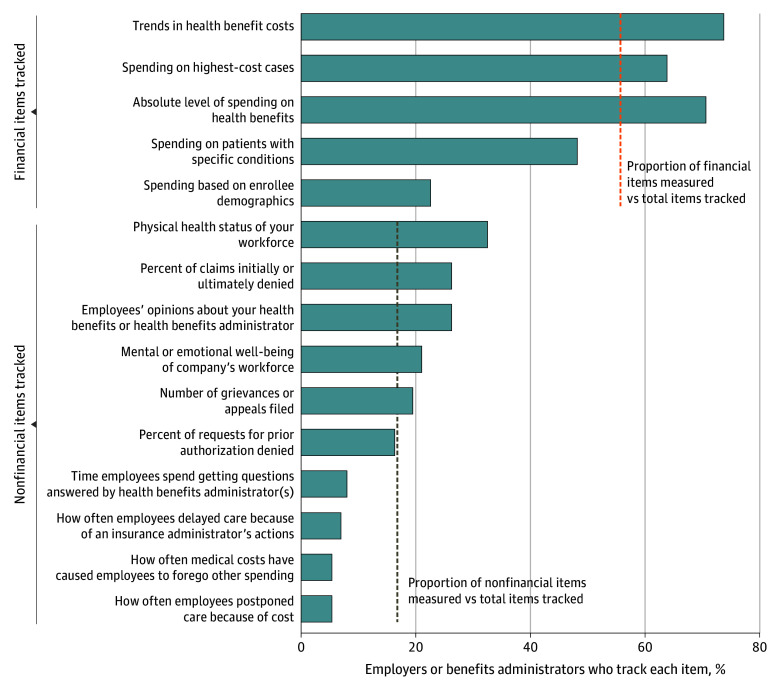
Information Tracked by Companies or Their Health Benefits Administrators These data were derived from the analysis of the Employee Healthcare Benefits Survey. Results are based on weighted data and are from question 19 which reads “Does your company, or your health benefits administrator on behalf of your company, track (in a deidentified way) any of the following: Please select all that apply.” Survey responses have been truncated to better fit the graph. For the full description of survey responses, see Supplement 1. Proportion of financial and nonfinancial items measured vs total items tracked was calculated as the mean for relevant items of the percentage of employers (or their benefits administrators) that track each item, represented by dashed lines.

Similarly, companies (or health benefits administrators on their behalf) are far more likely to track health spending than other information (question 19; Figure 3). The percentage of companies that track 5 categories related to health spending ranged from 125 (74%) for trends in health benefit costs to 38 (22%) for spending based on enrollee demographics, with 109 (64%) tracking spending on the highest cost cases, for a mean (SD) of 56% (21%) across the 5 categories provided. The mean (SD) percentage increases to 64% (11%) if spending information based on enrollee demographics was excluded.

The percentage of companies that track the 10 nonfinancial measures ranged from 55 (33%) for physical health status of the workforce to 10 (6%) for how often their employees postponed care, such as filling a prescription due to cost, or incurred medical costs that caused them to forego other spending. Given the prominence of this issue of foregoing care because of cost, ignoring this outcome is striking. Only 14 (8%) tracked time employees spent having questions answered, and 12 (7%) tracked how often employees delayed receiving care because of an insurance company’s actions. The mean (SD) percentage of nonfinancial health information tracked by companies was 17% (10%) across the 10 categories examined. Furthermore, while only 10 companies (6%) did not track any of the 5 financial measures, 58 (34%) did not track any of the 10 nonfinancial measures.

The mean (SD) number of financial items tracked per respondent was 2.8 (1.5) (of 5 total items, 56% [21%]), and the mean number of nonfinancial items tracked were 1.7 (2.0) (of 10 total items, 17% [3%]), a significant difference in the proportion of financial vs nonfinancial measures tracked (paired *t* test *P* < .001) (eTable 5, question 19 in Supplement 1).

### What Factors Relate to Companies’ Financial Focus

As expected, multivariable regression modeling of the paired differences in financial vs nonfinancial proportions identified very few organizational characteristics related to information included in the benefits administrator’s report (questions 11 and 12), overall tracking of benefits information by the company (question 19), and no characteristics related to consulting tasks (question 6), recipient of savings (question 23), or criteria for selecting a benefits administrator (question 9) (Table 2). Of 37 comparisons, 6 proportions (16%) differed significantly, with differences in proportions no greater than 0.22. The use of a benefits consulting firm was significantly associated with an increased emphasis on financial aspects of employee health benefits, both for the information included in the benefits administrator’s report and for benefits information tracked by the company. Specifically, the proportion of financial items was on average 0.22 greater than the proportion of nonfinancial items included in the report (*P* = .001), and the proportion of financial items tracked was on average 0.19 greater than the proportion of nonfinancial items tracked (*P* < .001). In contrast, greater frequency in soliciting employee feedback about their health benefits related to significantly less emphasis on financial matters for both the benefits administrator’s report and overall tracking of health information. The proportion of financial items was on average 0.05 less than the proportion of nonfinancial items for both items included in the report and items tracked (*P* = .02 and *P* = .001, respectively). Midsize companies (100-999 employees) compared to smaller companies (50-99 employees) and companies that use a benefits administrator focused more on financial matters in tracking health benefits information. The proportion of financial items tracked exceeded the proportion of nonfinancial items tracked on average by 0.10 (*P* = .049) for midsize companies relative to smaller companies, and by 0.15 (*P* = .01) for companies that use a benefits administrator compared to those that do not.

**Table 2.** Multivariable Regressions for (Paired) Difference in the Relative Number (Proportion) of Items or Composite Score^a^

Question No.	Variable	Consulting tasks (question 6)		Benefits administrator report (questions 11-12)		Benefits information tracked (question 19)		Savings recipient (question 23)		Benefits administrator criteria (question 9)	
		Estimate (95% CI)	*P* value	Estimate (95% CI)	*P* value	Estimate (95% CI)	*P* value	Estimate (95% CI)	*P* value	Estimate (95% CI)	*P* value
NA	Intercept	0.46 (0.35 to 0.57)	<.001	0.33 (0.18 to 0.48)	<.001	0.17 (0.04 to 0.31)	.01	0.18 (−0.005 to 0.36)	.06	0.76 (0.53 to 0.99)	<.001
3 (Relative to size 50-99 employees)	Size 100-999	0.02 (−0.10 to 0.13)	.74	0.09 (−0.07 to 0.25)	.28	0.10 (<0.001 to 0.21)	.049	−0.05 (−0.20 to 0.10)	.50	−0.13 (−0.38 to 0.12)	.31
	Size 1000-9999	0.04 (−0.18 to 0.27)	.69	0.02 (−0.26 to 0.30)	.89	0.11 (−0.09 to 0.30)	.29	−0.17 (−0.46 to 0.13)	.26	0.14 (−0.37 to 0.66)	.58
	Size ≥10 000	0.04 (−0.37 to 0.45)	.84	−0.13 (−0.69 to 0.43)	.65	−0.08 (−0.49 to 0.32)	.68	0.12 (−0.57 to 0.81)	.74	0.02 (−0.95 to 0.99)	.97
4	Self-insured	−0.07 (−0.20 to 0.05)	.26	−0.04 (−0.19 to 0.11)	.59	−0.03 (−0.14 to 0.08)	.61	−0.05 (−0.22 to 0.11)	.52	−0.03 (−0.31 to 0.25)	.83
5	Benefits consultant	NA	NA	0.22 (0.09 to 0.35)	.001	0.19 (0.10 to 0.29)	<.001	0 (−0.13 to 0.12)	.94	−0.11 (−0.32 to 0.09)	.28
7	Benefits administrator	−0.01 (−0.12 to 0.10)	.90	NA	NA	0.15 (0.04 to 0.26)	.01	−0.11 (−0.26 to 0.04)	.16	NA	NA
27	Mission references health	0.01 (−0.08 to 0.11)	.79	−0.04 (−0.16 to 0.08)	.48	−0.01 (−0.10 to 0.08)	.77	−0.08 (−0.20 to 0.04)	.18	−0.19 (−0.39 to 0.02)	.08
28	Strategic plan references health	−0.09 (−0.21 to 0.03)	.14	0.06 (−0.08 to 0.20)	.40	0.05 (−0.05 to 0.15)	.28	0 (−0.15 to 0.15)	.96	0.12 (−0.14 to 0.37)	.36
29/31/33	Culture of health	−0.01 (−0.06 to 0.04)	.74	0.06 (−0.02 to 0.13)	.15	0 (−0.05 to 0.05)	.98	−0.05 (−0.12 to 0.02)	.16	−0.11 (−0.23 to 0.01)	.07
26	Employee feedback	−0.02 (−0.05 to 0.01)	.29	−0.05 (−0.09 to −0.01)	.02	−0.05 (−0.08 to −0.02)	.001	−0.03 (−0.07 to 0.01)	.19	0.03 (−0.04 to 0.10)	.37
Model statistics	Observations, No.	146		105		188		199		182	
	*F* statistic	1.02		3.38		4.46		1.79		1.65	
	*P* value	.43		.001		<.001		.06		.11	
	*R* ^2^	6.3%		24.3%		20.1%		8.7%		7.9%	
	Adjusted *R*^2^	0.1%		17.1%		15.6%		3.9%		3.1%	

Abbreviation: NA, not applicable.

^a^ Based on weighted data. Survey item text follows: question 9, “On a scale of 0 to 5, when 0 means completely unimportant and 5 means extremely important, how important are the criteria in your company’s choice of health benefits administrator?”; question 6, “Which of the following, if any, does the health benefits consulting firm do for you?”; questions 11/12 (for companies receiving an annual report on the performance of their health plan from their health benefits administrator), “Which, if any, of the following spending/user experience information is included in the report?”; question 19, “Does your company, or your health benefits administrator on behalf of your company, track (in a deidentified way) any of the following?”; and question 23, “What does your company do with savings, if any, on health benefits?” For the full description of survey responses and composition of composites, see Figures 1-3 and refer to eAppendix in Supplement 1 section.

The analysis provided no evidence of any associations with self-insurance; reference to employee health and well-being in the company’s mission statement or business objectives; having a formal, written, strategic plan for health and well-being that covers at least 2 of the 4 domains of health (employee, environment, consumer, community); or actions to promote a culture of health (ie, having a wellness program, offering monetary rewards for healthy lifestyles, and undertaking initiatives, such as housing, financial, or tuition assistance to increase employee access to services promoting health and well-being). The analysis also did not reveal any factors significantly associated with financial vs nonfinancial criteria in choosing a benefits administrator (question 9), tasks performed by a consulting firm (question 6), or recipient of any savings (question 23).

## Discussion

In a nationally representative survey of employers providing health benefits, we showed that financial and monetary indicators received the overwhelming focus of measurement, benefits consulting firms also overwhelmingly focus on financial measures, and financial indicators are emphasized most strongly in decision-making about which health benefits administrators to use. This emphasis on the financial aspects of health plans is heightened among companies that use a benefits consulting firm but otherwise is not affected by factors that might plausibly influence the extent of financial emphasis, such as size (number of employees) or whether employers regard promoting health and well-being as part of their mission. Companies that more frequently seek employee feedback about their health benefits experience paid less attention to financial factors. The frequency of seeking employee feedback probably assesses an employer’s emphasis on the employee experience which, naturally, would cause employers to pay attention to the dimensions of that experience.

Financial measures and criteria dominate the oversight and decisions about health plans, while there is relative neglect of other indicators of health plan performance, such as the member experience, time spent negotiating with health benefits administrators, and overall employee behavioral and physical health. That this emphasis is largely independent of many explanatory factors indicates that employers’ emphasis on financial over nonfinancial aspects of health care has become truly institutionalized, that is, structures and rules have become widely accepted, reasonably stable over time, and largely independent of efficiency or other technical considerations.^9^ Emphasis on financial indicators of performance has become taken for granted and widely adopted without much consideration of anything other than “that is what employers do.”^10^

We note that such emphasis on the financial dimensions of health plans is not inevitable; decisions about which administrators to use and what to measure could include not only financial criteria but also important nonfinancial criteria such as member experience and access to care. Research has demonstrated that employee problems in interacting with benefits administrators about claims and authorizations are pervasive,^11^ absorb an economically significant amount of time, and that administrative burden adversely affects employee engagement and job satisfaction.^12^ Difficulties in accessing care because of cost, even for insured employees, has been a focus of both Gallup^13^ and KFF^14^ reports because of the empirical research relating access to care to health outcomes and because a significant fraction of the US population delays obtaining some form of care (physician visits and prescriptions).

### Limitations

The results of this study were gleaned from a cross-sectional survey, so we cannot claim causality. We also obtained a relatively low response rate leaving room for selection bias; we used recommended techniques for increasing response, and our response rate of more than 20% is not unusual for surveys of this type.^15^ Respondents did not differ from nonrespondents on known characteristics. We necessarily asked questions about a finite number of measures; we do not know if employers are measuring other things not queried in our survey. We also asked more questions about nonfinancial than financial measures; research suggests this would result in bias toward nonfinancial measures if counted.^16^ We sought to provide comparability by contrasting proportions of financial measures to proportions of nonfinancial measures; however, this approach results in higher proportions for constructs with fewer available options and thus may bias results toward financial measures.

## Conclusions

The data we present in this survey study likely reflect the taken-for-granted emphasis by employers on financial measures of health plan performance, to the neglect of other ways of assessing health plans. What we found may indicate the institutionalization of the prioritization of financial criteria in benefits administration. Because measures matter, employers’ emphasis on financial aspects may contribute to the problems observed in health benefits administration and health plan performance.

Health benefits plans exhibit numerous problems, ranging from employee dissatisfaction to problematic access to health care. As measurement is important in focusing attention and efforts, employers could and should act to incorporate a much broader and more comprehensive set of measures assessing how health plans operate. By expanding what they manage and measure beyond costs, employers might help expand attention to other dimensions of health plan performance implicating employee health and well-being.

## Notes

Supplement 1. eAppendix. Employee Health Care Benefits Survey eMethods. Methodology Report eTable 1. Sample Characteristics eTable 2. Q9: Financial and Non-Financial Criteria for Choosing a Health Benefits Administrator, Items and Composite Means eTable 3. Q6: Financial and Non-Financial Actions by Health Benefits Consulting Firms, Items and Composite Means eTable 4. Q11/12: Financial and Non-Financial Information Included in the Health Benefits Administrator’s Annual Report, Items and Composite Means eTable 5. Q19: Financial and Non-Financial Items Tracked, Items and Composite Means eTable 6. Q23: Uses of Savings that Benefit the Company and Employees, Items and Count Sample Percentages eReferences

Supplement 2. Data Sharing Statement
