# Supplementary material for: Emphasis on Financial vs Nonfinancial Criteria in Employer Benefits’ Measurements
Source: JAMA Health Forum. 2025 Jan 31;6(1):e245229. doi: 10.1001/jamahealthforum.2024.5229 (PMC11786228; doi:10.1001/jamahealthforum.2024.5229)
Supplement: Supplement 1. — eAppendix. Employee Health Care Benefits Survey eMethods. Methodology Report eTable 1. Sample Characteristics eTable 2. Q9: Financial and Non-Financial Criteria for Choosing a Health Benefits Administrator, Items and Composite Means eTable 3. Q6: Financial and Non-Financial Actions by Health Benefits Consulting Firms, Items and Composite Means eTable 4. Q11/12: Financial and Non-Financial Information Included in the Health Benefits Administrator’s Annual Report, Items and Composite Means eTable 5. Q19: Financial and Non-Financial Items Tracked, Items and Composite Means eTable 6. Q23: Uses of Savings that Benefit the Company and Employees, Items and Count Sample Percentages eReferences [file jamahealthforum-e245229-s001.pdf]

Supplemental Online Content

Pfeffer J, Olsen E, Singer SJ. Emphasis on financial vs nonfinancial criteria in employer benefits' measurements. *JAMA Health Forum.* 2025;6(1):e245229. doi:10.1001/jamahealthforum.2024.5229

eAppendix. Employee Health Care Benefits Survey

eMethods. Methodology Report

eTable 1. Sample Characteristics

eTable 2. Q9: Financial and Non-Financial Criteria for Choosing a Health Benefits Administrator, Items and Composite Means

eTable 3. Q6: Financial and Non-Financial Actions by Health Benefits Consulting Firms, Items and Composite Means

eTable 4. Q11/12: Financial and Non-Financial Information Included in the Health Benefits Administrator's Annual Report, Items and Composite Means

eTable 5. Q19: Financial and Non-Financial Items Tracked, Items and Composite Means

eTable 6. Q23: Uses of Savings that Benefit the Company and Employees, Items and Count Sample Percentages

eReferences

This supplemental material has been provided by the authors to give readers additional information about their work.

© 2025 Pfeffer J et al. *JAMA Health Forum.*

eAppendix. Employee Healthcare Benefits Survey

Welcome to the Employee Healthcare Benefits Survey

INSTRUCTIONS: Thank you for your help with this important study. With support from the Robert Wood Johnson Foundation, researchers at the Stanford Graduate School of Business and Stanford University School of Medicine are fielding this survey to understand how U.S.-based companies manage healthcare benefits for their employees. All responses are private and confidential. Results will be analyzed only in the aggregate and no individual responses will be reported. Your participation is critical to inform these findings, but this survey is voluntary. You have the right to refuse to answer questions.

The survey should be completed by the Human Resources or Health Benefits administrator, or the member of the C-suite that is most knowledgeable about health benefits decisions.

Please read each question carefully. Using a blue or black pen, mark the box next to the appropriate response or responses as indicated. Please answer all questions unless directed otherwise. It should take you about 15 minutes to complete. Once you have completed the survey, please return it in the enclosed postage-paid envelope.

If you have any questions about the survey, please call Akilah Evans-Pigford at 1-800-633-1986, Ext. 4314 or email at aevans.pigford@ssrs.com.

SECTION I: GENERAL INFORMATION

This survey will refer to the following terms:

- A ***health benefits consulting firm*** advises companies about health benefits / insurance options for their employees. In some instances, they provide actuarial estimates of cost assuming different plan designs. Some examples include Hewitt, Aon, Mercer, Willis Towers Watson, or a smaller entity in that business.
- A ***health benefits administrator*** is contracted by a company to manage its employees' health benefits and the day-to-day operations of the health plan on behalf of the organization. Some examples include Anthem, Blue Cross Blue Shield, Cigna, United Health, Humana.
- An ***HR or benefits role*** is one in which you interact with your company's health benefits administrator or a health benefits consulting firm.
- A ***health plan*** is an insurance plan that is used to cover costs associated with medical care.

First, we'd like to ask you questions about your background.

1. During your professional career, have you ***ever*** worked in one of the following roles or types of organization? **Please select all that apply.**

   ❑ A *health benefits consulting firm*
   ❑ A *health benefits administrator*
   ❑ An *HR or benefits role*
   ❑ A *health plan*
   ❑ None of the above

© 2025 Pfeffer J et al. *JAMA Health Forum.*

Now, we'd like to ask about your current role.

2. What is your current role in your company? **Please select all that apply.**

❑ An *HR or benefits role* in which you interact with your company's health benefits administrator or a health benefits consulting firm.
❑ C-Suite leader within a company
❑ Other (please specify) ____

3. Including yourself, about how many employees does your company or organization have, including both full-time and part-time? This would be at all locations, and, if you are a franchise of a national chain, please answer for just the locations you own or manage.

❑ Less than 50 ⟶ How many employees does your company or organization have? ________ **# employees**
❑ 50 to 99
❑ 100 to 999
❑ 1000 to 9,999
❑ 10,000 or more

Now, we'd like to ask you questions about your company. Companies may self-insure their employees' health benefits by paying directly for all medical claims or they may fully-insure by purchasing health benefits for their employees through an insurance company. Your plan is still considered fully insured if your company purchases stop-loss coverage.

4. For your employee health benefits, is your company self-insured or fully insured?

❑ Self-insured
❑ Fully insured
❑ Not sure

SECTION II: HEALTH BENEFITS CONSULTING INTERACTIONS

Now, we'd like to ask you questions about your company's interaction with health benefits consultants.

5. Does your company regularly use a health benefits consulting firm, such as Hewitt, Aon, Mercer, Willis Towers Watson, or a smaller entity in that business?

❑ Yes ⟶ **Continue to Question 6**
❑ No
❑ Not sure ⟶ **Skip to Question 7**

© 2025 Pfeffer J et al. *JAMA Health Forum.*

6. Which of the following, if any, does the health benefits consulting firm do for you? **Please select all that apply.**

Health Benefits Consulting Tasks	
Advises on the quality or makes recommendations for a health benefits administrator	❑
Provides cost estimates, including projections of healthcare costs	❑
Makes recommendations about health plan design, including copayments, deductibles, etc.	❑
Provides information on the effects of health plan design changes on employee behavior	❑
Helps monitor employee physical health trends in aggregate, such as healthy days at work	❑
Helps monitor employee mental health trends in aggregate	❑
Compares your health benefits with relevant benchmarks	❑
Compares your health care costs and service utilization with relevant benchmarks	❑

SECTION III: HEALTH BENEFITS ADMINISTRATOR

Now, we'd like to ask about your company's health benefits administrator. *If your company works with more than one health benefits administrator, please answer the following questions based on the one that plays the largest role. Refer to this health benefits administrator in the remainder of this survey.*

7. Does your company regularly use one or more health benefits administrators, such as UnitedHealth Group, Anthem, Humana, Blue Cross Blue Shield, CVS Health, Kaiser Permanente?

❑ Yes, more than one
❑ Yes, one
Continue to Question 8

❑ No
❑ Not sure
Continue to Question 17

8. Does your health benefits administrator also directly offer medical care services to your employees?

❑ Yes
❑ No
❑ Not sure

9. On a scale from 0 to 5, where 0 means completely unimportant and 5 means extremely important, how important are the following criteria in your company's choice of a health benefits administrator? **Please record a response for each item below.**

Administrator Criteria	0 – Completely unimportant	1	2	3	4	5 – Extremely important	Not Sure
Cost or price of the administration	❑	❑	❑	❑	❑	❑	❑
Kind of payment (i.e., flat fee, % of claims, other)	❑	❑	❑	❑	❑	❑	❑
Hours of telephone availability to employees	❑	❑	❑	❑	❑	❑	❑

© 2025 Pfeffer J et al. *JAMA Health Forum.*

On a scale from 0 to 5, where 0 means completely unimportant and 5 means extremely important, how important are the following criteria in your company's choice of a health benefits administrator? **Please record a response for each item below.**

Administrator Criteria	0 – Completely unimportant	1	2	3	4	5 – Extremely important	Not Sure
Member satisfaction	❑	❑	❑	❑	❑	❑	❑
Quality ratings, such as those on HealthCare.gov	❑	❑	❑	❑	❑	❑	❑
Overall size	❑	❑	❑	❑	❑	❑	❑
Prestige or brand recognition	❑	❑	❑	❑	❑	❑	❑
Market share in locations where you operate	❑	❑	❑	❑	❑	❑	❑
Ease of use of the administrator's website	❑	❑	❑	❑	❑	❑	❑
Ease of locating in-network doctors, labs, and hospitals	❑	❑	❑	❑	❑	❑	❑
Pricing of services for comparable doctors, laboratories, and hospitals	❑	❑	❑	❑	❑	❑	❑
Employee health outcomes	❑	❑	❑	❑	❑	❑	❑
Prior relationships of company employees with the health benefits administrator	❑	❑	❑	❑	❑	❑	❑
Licensed carrier	❑	❑	❑	❑	❑	❑	❑

10. Does your health benefits administrator provide an annual report on the performance of your health plan?

❑ Yes → **Continue to Question 11**
❑ No
❑ Not sure } **Skip to Question 13**

11. Which, if any, of the following spending information is included in the report? **Please select all that apply.**

Health Care Spending Information Reported	
Total health care spending	❑
Trend in spending over the past several years	❑
Spending on highest-cost cases, where identity of the case is not disclosed	❑
Spending on patients with specific conditions, such as patients with diabetes, hypertension, etc.	❑
Spending based on enrollee demographics, such as income, gender, and race/ethnicity	❑
None of the above	❑
Other (please specify) ____	❑
Not sure	❑

12. Which, if any, of the following user experience information is included in the report? **Please select all that apply.**

© 2025 Pfeffer J et al. *JAMA Health Forum.*

User Experience Information Reported	
Amount of time employees spend getting questions answered by health benefits administrator(s)	❑
Percent of claims initially or ultimately denied	❑
Percent of requests for prior authorization denied	❑
Number of grievances or appeals filed	❑
How often employees postponed filling a prescription, visiting a doctor, or having a medical procedure because of cost	❑
How often employees delayed filling a prescription, visiting a doctor, or having a medical procedure because of an insurance administrator's actions, such as denying authorization	❑
Measures of employee satisfaction with their health benefits or your health benefits administrator	❑
None of the above	❑
Not sure	❑

13. In the last year, did you ask your health benefits administrator to track any new metrics for your company?

❑ Yes
❑ No
❑ Not sure

14. In the last year, did you request that your health benefits administrator make any changes to the medical services covered by your company's health benefits?

❑ Yes
❑ No
❑ Not sure

15. In the last 5 years, how many times did you review your health benefits administrator?

❑ Never
❑ Once
❑ Twice
❑ Three times
❑ Four times
❑ Five or more times
❑ Not sure

16. In the last 5 years, how many times did you change your health benefits administrator?

❑ Never
❑ Once
❑ Twice
❑ Three times
❑ Four times
❑ Five or more times
❑ Not sure

© 2025 Pfeffer J et al. *JAMA Health Forum.*

SECTION IV: YOUR COMPANY

Now, we'd like to ask you about your company.

17. In your company, in which of the following decisions, if any, does your Chief Executive Officer typically participate? **Please record a response for each item below**.

Chief Executive Officer Decisions	Yes	No	Not sure
Copay or coinsurance levels for employees	❑	❑	❑
Deductible limit or deductible options for employees	❑	❑	❑
Levels of company versus employee contribution to health premiums	❑	❑	❑
Health plan design	❑	❑	❑
Choice or evaluation of health benefits administrator	❑	❑	❑
Choice of which benefits consultants to engage with	❑	❑	❑
Incentives for employee engagement in health improvement activities	❑	❑	❑

18. In your company, in which of the following decisions, if any, does your Chief Financial Officer, or equivalent, typically participate? **Please record a response for each item below**.

Chief Financial Officer Decisions	Yes	No	Not sure
Copay or coinsurance levels for employees	❑	❑	❑
Deductible limit or deductible options for employees	❑	❑	❑
Levels of company versus employee contribution to health premiums	❑	❑	❑
Health plan design	❑	❑	❑
Choice or evaluation of health benefits administrator	❑	❑	❑
Choice of which benefits consultants to engage with	❑	❑	❑
Incentives for employee engagement in health improvement activities	❑	❑	❑

19. Does your company, or your health benefits administrator on behalf of your company, track (in a deidentified way) any of the following: **Please select all that apply**.

Company Health Benefits Information Tracked	
Physical health status of your workforce, such as self-reported health, incidence of diabetes or other diseases of your company's workforce	❑
Mental or emotional well-being of your company's workforce	❑
Absolute level of spending on health benefits	❑
Trends in health benefit costs	❑

© 2025 Pfeffer J et al. *JAMA Health Forum.*

Does your company, or your health benefits administrator on behalf of your company, track (in a deidentified way) any of the following: **Please select all that apply**.

**Company Health Benefits Information Tracked**	
Spending on highest-cost cases, where identity of the case is not disclosed	❑
Spending on patients with specific conditions, such as patients with diabetes, hypertension, etc.	❑
Spending based on enrollee demographics, such as income, gender, race/ethnicity	❑
Your employees' opinions about your health benefits or health benefits administrator	❑
Amount of time employees spend getting questions answered by health benefits administrator(s)	❑
Percent of claims initially or ultimately denied	❑
Percent of requests for prior authorization denied	❑
Number of grievances or appeals filed	❑
How often employees delayed filling a prescription, visiting a doctor, or having a medical procedure because of an insurance administrator's actions (e.g., denying authorization)	❑
How often employees postponed filling a prescription, visiting a doctor, or having a medical procedure because of cost	❑
How often medical costs have caused employees to forego other spending	❑
Not sure	❑

20. Within your company, who, if anyone, has accountability for performance on the following?
Please record a response for each item below.

**Performance Accountability**	**Human Resources Administrator**	**Company leader outside of Human Resources**	**Someone else**	**No one**	**Other**	**Not Sure**
Physical health status of your workforce, such as self-reported health, incidence of diabetes or other diseases of your company's workforce	❑	❑	❑	❑	❑________	❑
Mental or emotional well-being of your company's workforce	❑	❑	❑	❑	❑________	❑
Absolute level of spending on health benefits	❑	❑	❑	❑	❑________	❑
Trends in health benefit costs	❑	❑	❑	❑	❑________	❑
Spending on highest-cost cases, where identity of the case is not disclosed	❑	❑	❑	❑	❑________	❑
Spending on patients with specific conditions, such as patients with diabetes, hypertension, etc.	❑	❑	❑	❑	❑________	❑

© 2025 Pfeffer J et al. *JAMA Health Forum.*

Within your company, who, if anyone, has accountability for performance on the following?
Please record a response for each item below.

Performance Accountability	Human Resources Administrator	Company leader outside of Human Resources	Someone else	No one	Other	Not Sure
Spending based on enrollee demographics, such as income, gender, race/ethnicity	❑	❑	❑	❑	❑________	❑
Your employees' opinions about your health benefits or health benefits administrator	❑	❑	❑	❑	❑________	❑
Amount of time employees spend getting questions answered by health benefits administrator(s)	❑	❑	❑	❑	❑________	❑
Percent of claims initially or ultimately denied	❑	❑	❑	❑	❑________	❑
Percent of requests for prior authorization denied	❑	❑	❑	❑	❑________	❑
Number of grievances or appeals filed	❑	❑	❑	❑	❑________	❑
How often employees delayed filling a prescription, visiting a doctor, or having a medical procedure because of an insurance administrator's actions (e.g., denying authorization)	❑	❑	❑	❑	❑________	❑
How often employees postponed filling a prescription, visiting a doctor, or having a medical procedure because of cost	❑	❑	❑	❑	❑________	❑
How often medical costs have caused employees to forego other spending	❑	❑	❑	❑	❑________	❑

21. What does your company do, if anything, to ensure that you are getting the best value available on the market for health benefits? **Please select all that apply below**.

Strategies for Ensuring Value of Health Benefits	
Conduct benchmark research to compare your plan with the plans of similar companies	❑
Request offers from multiple health benefit administrators	❑
Negotiate the price directly with the health benefit administrator	❑
Contract directly with service providers	❑

© 2025 Pfeffer J et al. *JAMA Health Forum.*

What does your company do, if anything, to ensure that you are getting the best value available on the market for health benefits? **Please select all that apply below**.

Strategies for Ensuring Value of Health Benefits	
Offer a health plan option that eliminates doctors and/or hospitals with higher-than-average charges unless there is proof that they provide higher than average quality of care?	❑
Other (please specify) ____	❑
Not sure	❑

22. What does your company do, if anything, to reduce spending on health benefits? **Please select all that apply below**.

Strategies for Reducing Health Benefit Expenditures	
Shift healthcare costs to employees through higher copays, premiums, coinsurance, or deductibles	❑
Require spousal surcharges for dependent spouses who have the option of enrolling in health insurance through another source	❑
Use tiered or narrow network plans	❑
Require assignment to primary care physician	❑
Require prior authorization	❑
Financially incentivize employees to enroll in health and wellness programs	❑
Financially incentivize employees to seek high-value care	❑
Other (please specify) ____	❑
Not sure	❑

23. What does your company do with savings, if any, on health benefits?

❑ Apply the savings directly to reduce employee cost sharing
❑ Add the savings back into employee paychecks
❑ Use the savings within your company's general fund
❑ Add the savings into shareholder earnings
❑ Not applicable
❑ Other (please specify) _______________________________
❑ Not sure

24. Does your company give 100% of savings from health plan options that cost less to employees that choose this option?

❑ Yes
❑ No
❑ Not sure

25. How often does your company review your health plan?

❑ Quarterly or more frequently
❑ Semi-annually
❑ Annually
❑ Every other year
❑ Less frequently than every other year
❑ Never
❑ Not sure

26. How often does your company request feedback from employees regarding their health benefits?

❑ Quarterly or more frequently
❑ Semi-annually
❑ Annually
❑ Every other year
❑ Less frequently than every other year
❑ Never
❑ We do not request feedback from employees
❑ Not sure

27. Does your company's mission or business objectives include reference to improving or maintaining employee health and well-being?

❑ Yes
❑ No
❑ Not sure

28. Does your company have a formal, written, strategic plan for health and well-being across at least some of these four domains—employee, environmental, consumer, and community?

❑ Yes, we have a plan addressing all four of these domains
❑ Yes, we have a plan across at least two of these domains
❑ No, we do not have such a plan
❑ Not sure

29. Does your company offer programs to promote employee health and wellness?

❑ Yes
❑ No
❑ Not sure

If you said yes at Question 29 above and your company offers health and wellness programs:

30. In the past five years, has your company conducted an assessment of the impact of your health and well-being programs on **employee** health, or not?

❑ Yes
❑ No
❑ Not sure

© 2025 Pfeffer J et al. *JAMA Health Forum.*

31. Does your company offer monetary rewards to your employees for healthy lifestyles, such as free or subsidized health or fitness club memberships, weight loss program memberships or fees, deductibles for treatment compliance or medication adherence?

❑ Yes
❑ No
❑ Not sure

If you said yes at Question 31 above and your company offers monetary awards for healthy lifestyles:

32. Does your company offer monetary rewards to your employees for any of the following?

Please select all that apply below.

❑ Free or subsidized health or fitness club memberships
❑ Weight loss program memberships or fees
❑ Deductibles for treatment compliance or medication adherence
❑ Other (please specify) ____
❑ Not sure

© 2025 Pfeffer J et al. *JAMA Health Forum.*

33. Does your company undertake initiatives, like housing, financial, or tuition assistance, to increase access to services for employees that would indirectly improve their health and wellbeing?

- ❑ Yes
- ❑ No
- ❑ Not sure

If you said yes at Question 33 above and your company undertake initiatives to increase access to services:

34. If yes, does your company undertake initiatives to address any of the following?

Please select all that apply below.

- ❑ Housing insecurity
- ❑ Financial insecurity (e.g., financial education)
- ❑ Tuition assistance
- ❑ Childcare
- ❑ Other (please specify) ____
- ❑ Not sure

If you said yes at Question 33 above and your company undertake initiatives to increase access to services:

35. Do you think the financial return on your company's investment in employee health initiatives is currently positive, neutral, or negative?

- ❑ Very positive
- ❑ Somewhat positive
- ❑ Neutral
- ❑ Somewhat negative
- ❑ Very negative
- ❑ Not sure

© 2025 Pfeffer J et al. *JAMA Health Forum.*

36. How attractive do you believe are the following company offerings to employees at your company? On a scale of 0-5 with 0 being the least attractive and 5 being the most attractive.

Importance of Company Offerings	0 – Least attractive	1	2	3	4	5 – Most attractive	Not Sure
Opportunity to work on the company's products or services	❑	❑	❑	❑	❑	❑	❑
Salary compared to other companies	❑	❑	❑	❑	❑	❑	❑
Health benefits	❑	❑	❑	❑	❑	❑	❑
Other benefits	❑	❑	❑	❑	❑	❑	❑
Perceived culture within the company	❑	❑	❑	❑	❑	❑	❑
Care for employee wellbeing or work life balance	❑	❑	❑	❑	❑	❑	❑
Perceived growth opportunities within the company	❑	❑	❑	❑	❑	❑	❑
The company's mission and purpose	❑	❑	❑	❑	❑	❑	❑

These last few questions are for classification purposes only and will help us to better understand your answers.

37. In the last year, what is the average sick time per employee in your company?

❑ Less than 3 days
❑ 3 to 5 days
❑ 6 to 10 days
❑ More than 10 days
❑ Not sure

38. What is average tenure of employees in your company?

❑ Less than a year
❑ 1 year but less than 3 years
❑ 3 years but less than 5 years
❑ 5 years but less than 10 years
❑ 10 years but less than 15 years
❑ 15 years or more
❑ Not sure

39. Last year, how much did your company spend on healthcare per employee?

❑ Less than $5,000
❑ $5,000 to less than $10,000
❑ $10,000 to less than $15,000
❑ $15,000 to less than $20,000
❑ $20,000 or more
❑ Not sure

© 2025 Pfeffer J et al. *JAMA Health Forum.*

40. Which of the following best describes your company or organization? Please select one.

❑ Manufacturing
❑ Agriculture
❑ Mining, extraction, oil
❑ Construction
❑ Transportation
❑ Retail Sales
❑ Banking, finance, insurance, real estate
❑ Other Services
❑ Food industry
❑ Media, communications
❑ Health care
❑ Education
❑ Something else (please specify) ____
❑ Not sure

If you indicated "Retail Sales", "Food Industry", or "Other Services"/ "Something Else" at Question 40 above and your organization or business is Retail Sales, Food Industry, or Other Services/ Something Else:

41. Please provide a little more detail on the industry category that your company/organization falls into. **Please select only one.**

❑ Business Services such as administrative or support, staffing, printing, advertising, consulting, etc.
❑ Personal Services such as hair salon, barber, nail salon, tax preparation, dry cleaning, child daycare, etc.
❑ Computer Software or Services
❑ Building services such as maintenance, security products & services, cleaning, landscaping services, etc.
❑ Professional, Scientific or Technical Services, including legal, medical, etc.
❑ Hospitality, such as hotels, motels, or other lodging places
❑ Restaurants, Eating and Drinking Places
❑ Arts, Entertainment and Recreation
❑ Automotive
❑ Other
❑ Not sure

© 2025 Pfeffer J et al. *JAMA Health Forum.*

CONCLUSION

Thank you very much for your response and participation in this survey. If you would like us to send you the results of the research once it is complete, please provide your email address here. We will not share this address or use it for any other purpose.

Email Address: ______________________________

❑ I do not wish to receive a copy of the findings.

If you have any questions about the survey, please contact the survey's Research Director, Akilah Evans-Pigford at 1-800-633-1986, Ext. 4314 or email at aevans.pigford@ssrs.com.

That completes the survey. To ensure that your survey reaches us, please return it in the enclosed postage-paid envelope to:

Survey of Employee Healthcare Benefits
SSRS | PO Box 5703 | Hopkins MN 55343-7063

© 2025 Pfeffer J et al. *JAMA Health Forum.*

METHODOLOGY REPORT

EMPLOYEE BENEFITS SURVEY

Prepared for Stanford Graduate School of Business and Stanford University School of Medicine

Prepared by:

Eran Ben-Porath

Akilah Evans-Pigford

MAY 2023

ssrs
research. refined.

Table of Contents

Overview........19
Questionnaire Design........19
Sample Design:........19
Data Collection........20
  Mailing and Recruitment........20
  Survey Administration Procedures........21
Response Rate........21
Data Processing and Integration........22
Weighting........22
  Post-Stratification Weighting (Raking)........22
  Weight Truncation........23
  Table 4: Sample Demographics Summary........23
  Effects of Sample Design on Statistical Analysis........23
Deliverables........23
APPENDIX........24

Overview

SSRS conducted the Employee Benefits Survey on behalf of the Stanford Graduate School of Business and Stanford University School of Medicine, between May 5, 2022 to April 25, 2023. The goal of the study was to survey human resource (HR) administrators about their experiences on managing healthcare benefits for their employees.

The study was conducted by mail (n=115), by phone(n=6), and online (n=130) with administrators at 251 qualifying businesses and organizations.

This report details the methodological components of the study: questionnaire design, sampling, data collection, data processing, weighting, and response rates.

Questionnaire Design

The questionnaire was developed by Stanford researchers in consultation with the SSRS project team. The questionnaire was designed initially as a hardcopy mail questionnaire. Once the mail-format was approved, the survey was programmed by SSRS into its Confirmit WebCATI software (into web and telephone modalities). Since hardcopy respondents cannot be programmatically forced to answer any question, the web questionnaire also allowed respondents to skip any questions they preferred not to answer with the exception of the screening questions that determined eligibility.

Extensive checking of the program was conducted to ensure the questionnaire appeared correctly in telephone and web modes. SSRS project staff ensured that skip patterns and question logic followed the design of the questionnaire for both modalities. The telephone mode was modified to include interviewer instructions. The web mode was programmed to accommodate multiple platforms such as laptops, cell phones, and tablets as well as different operating systems.

Revisions based on the pilot interviewing conducted between June 6- July 14, 2022; resulted in minor adjustments to the questionnaire that were applied to the CATI and Web modalities programming before the main phase of the study commenced on October 27,2022.

Sample Design:

The human resources administrators were sampled from a Dun and Bradstreet (D&B) database of businesses provided by SSRS. This database included contact information such as mailing address, telephone number, target job title, contact name along with information about the business or organization, such as number of employees and industry.

The initial phase (Pilot Study) of the Employee Benefits Survey consisted of a random sample of 200 businesses and organizations generated from the D&B Listing. The sample consisted of businesses and organizations who employ at least 50 employees across all its sites, is a single-site, headquarters or sub-headquarters of a multi-site; excluding businesses affiliated with government or public administration. The sample was drawn to be geographically representative of businesses in the U.S. and stratified by employee sizes (50-99, 100-499, 500+).

The main phase (Main Study) of the Employee Benefits Survey consisted of a random sample of 1,118 businesses and organizations generated from the D&B database (including 1,000 initial sample and 118 additional sample records procured in November 2022). The sample consisted of businesses and organizations who employ at least 50 employees across all its sites, is a single-site, headquarters or sub-headquarters of a

multi-site; excluding businesses affiliated with government or public administration. The sample was drawn to be geographically representative of businesses in the U.S. and stratified by employee sizes (50-99, 100-499, 500+).

Data Collection

Mailing and Recruitment

The study followed a schedule designed to maximize the likelihood of reaching HR administrators who would participate in the survey. The pilot and main phases involved multiple steps including identifying target respondents; multiple mail contacts; additional facility-level research; multiple additional contacts including telephone interviewing during the pilot phase of the study.

Identifying target respondents: SSRS researchers via online research and phone follow-up, identified in total names initially matched to 16 businesses and organizations for pilot phase (the remaining 161 were updated) and 425 for main phase (the remaining 575 were updated).

During the identification process, each business or organization was researched to validate and/or update if needed the following information:

- Business/Organization Validity
- Business/Organization Name
- Business/Organization Mailing Address
- Accuracy of Job Title and Contact Name on Sample
    - If the job title and contact name on sample was not accurate or desired job title, further research was conducted including search and locator process for a replacement contact with priority for Human Resources administrators that were mid-level employees who would have a higher probability of completing the survey including titles like Benefits Manager, Benefits Coordinator, Human Resources Manager. If contacts available did not fall into those targeted areas; further exploration was made using job titles that would be Human Resources C- Suite level employees including titles like Chief People Officer, Chief Human Resources Officer, Director if Human Resources to replace original contact and job title.

Multiple mail contacts: The following contact attempts were made:

- Invitation (including URL to web survey) with consent document, hardcopy questionnaire, business return envelope and $75 non-contingent incentive check
- Reminder Letter (including URL to the web survey) with consent document
- Reminder Letter (including URL to the web survey) with consent document, hardcopy questionnaire, business return envelope and re-issued $75 incentive check

The invitation letters were printed on Stanford Graduate School of Business and Stanford University School of Medicine letterhead and explained the purpose of the study and its importance. Respondents were encouraged to go online and complete the survey through a password-protected designated website (www.stanfordhealthstudy.com). Each respondent was assigned a unique 6-character password. For those preferring to respond in pencil-and-paper format, hardcopy questionnaires and business-reply envelopes were included with the original mailing. Sample recruiting materials can be found in the Appendix.

Additional research: To improve the likelihood of reaching the qualified respondent another round of online and phone verification with the businesses and organizations was conducted by SSRS.

Computer Assisted Telephone Interviewing (CATI): SSRS interviewers conducted 6 Pilot Study phone interviews with respondents who had not completed by web or hardcopy between June 6 to July 14, 2022.

Survey Administration Procedures

For the Employee Benefits Survey, the data collection schedule was as follows:

Table 1: Pilot Study Schedule:

Touchpoint	Date
Invitation with Incentive via Federal Express	May 5, 2022
Initial Reminder via USPS	May 25, 2022
CATI Outbound Field Starts	June 6, 2022
Final Reminder with Re-issued Check via Federal Express	June 29, 2022
CATI Outbound Field Ends	July 14, 2022

Table 2: Main Study Schedule:

Touchpoint	Date
Invitation with Incentive via Federal Express	October 27, 2022
Initial Reminder via USPS	November 10, 2022
CATI Outbound Reminders Start	November 11,2022
Additional Verification Outreach Start	November 16, 2022
CATI Outbound Reminders End	December 21, 2022
Additional Verification Outreach End	December 30, 2022
Invitation with Incentive via Federal Express(revised contacts)	January 24, 2023
Final Reminder with Re-issued Check via Federal Express (contacts with no changes)	January 24, 2023
Email reminders	February 6, 2023
Initial Reminder via USPS(revised contacts)	February 8, 2023
Invitation with Incentive via Federal Express(replaced contacts)	February 9, 2023
Invitation with Re-issued Incentive via Federal Express(revised contacts)	March 23, 2023
Final Reminder with Re-issued Check via Federal Express (replaced contacts)	April 11, 2023

Mean survey length was 16.8 minutes for CATI and Web completes combined.

Response Rate

The response rate for this study was calculated to be 22%. Table 3 provides a full disposition of the sample selected for this survey.

Table 3: Sample Dispositions

Eligible, Interview (Category 1)	Pilot	Main	Total
Complete	57	194	251
Eligible, non-interview (Category 2)			
Refusal (remove from list)	48	12	60
Incomplete	0	11	11
Unknown eligibility, non-interview (Category 3)			
No response (including those who did not click on the link)	80	757	837
Not eligible (Category 4)			
Non-Deliverable	9	135	144
Respondent reached and ineligible	3	6	9
Respondent reported as ineligible	3	3	6
TOTAL Sample	200	1,118	1,318

Data Processing and Integration

SSRS implemented several quality assurance procedures in data file preparation and processing. In addition to extensive testing of the telephone and web programming prior to the launching data collection, shortly after fielding began survey data were carefully checked for accuracy, completeness, and non-response to specific questions so that any issues could be identified and resolved if applicable.

The data file programmer implemented a "data cleaning" procedure in which survey skip patterns were created in order to ensure that all questions had the appropriate numbers of cases. This procedure involved a check of raw data by a program that consisted of instructions derived from the skip patterns designated on the questionnaire. The program confirmed that data were consistent with the definitions of codes and ranges and matched the appropriate bases of all questions.

A series of data quality checks were run on the final data. These checks included:

- Item non-response. Cases with more than 25% item non-response were flagged and reviewed
- Survey length. Cases with a length under 7 minutes were flagged and reviewed
- Cases would have been removed from the data if they failed one or more of the above quality checks. Based on this criterion, 6 cases were removed.

Weighting

The data were weighted to match the sample of respondents to the D&B database.

Post-Stratification Weighting (Raking)

The sample was weighted to match the demographic profile of businesses with over 50 employees. Weighting parameters were obtained from D&B. Weighting was done using iterative proportional fitting (or "raking") using SPSSINC RAKE, an SPSS extension module that simultaneously balances the distributions of all variables using the GENLOG procedure.

The parameters used for raking were industry, company size, and census region. Table 3 details the population parameters, unweighted percentage, and weighted percentage for each category used in weighting.

Weight Truncation

Weights were trimmed at the 2nd and 98th percentiles to prevent individual cases from having too much influence on the final results.

Table 4: Sample Demographics Summary

		Benchmark	Unweighted	Weighted
**Industry**	Manufacturing	16.0%	17.1%	16.1%
	Agriculture	1.2%	2.4%	1.2%
	Mining, extraction, oil	0.8%	1.6%	0.8%
	Construction	7.3%	6.0%	7.3%
	Transportation	6.0%	4.0%	5.8%
	Retail Sales	12.7%	10.8%	12.7%
	Banking, finance, insurance, real estate	8.0%	7.6%	8.0%
	Other Services	41.1%	46.2%	41.2%
	Wholesale Trade	7.0%	4.4%	6.8%
**Region**	NE	20.1%	17.5%	20.1%
	MW	23.7%	27.9%	23.8%
	S	34.8%	35.9%	34.8%
	W	21.4%	18.7%	21.3%
**Employees**	50-99	50.1%	35.9%	50.1%
	100-499	43.8%	26.3%	43.8%
	500+	6.1%	37.8%	6.1%

Effects of Sample Design on Statistical Analysis

Specialized sampling designs and post-data collection statistical adjustments require analysis procedures that reflect departures from simple random sampling. SSRS calculates the effects of these design features so that an appropriate adjustment can be incorporated into tests of statistical significance when using these data. This design (*deff*) represents the loss in statistical efficiency that results from a complex sample designs and systematic non-response. SSRS calculates the composite design effect for a sample of size n, with each case having a weight, w, as:

$$deff = \frac{n \sum w^2}{(\sum w)^2}$$

The design effect for this survey was 1.64. The corresponding margins of sampling error (MoSE) is +/- 7.9%.

Deliverables

Final deliverables for this study were as follows:

- Weighted SPSS file
- Methodology Report

APPENDIX

Sample invitation as follows:

Date

RECIPIENT FULL NAME

RECEIPENT JOB TITLE

ORGANIZATION NAME

RECIPIENT ADDRESS

CITY, STATE, ZIP CODE

Dear [INSERT NAME],

With support from the Robert Wood Johnson Foundation, researchers at the Stanford Graduate School of Business and Stanford University School of Medicine are hoping to better understand how U.S.-based companies manage healthcare benefits for their employees. The results of this survey will provide valuable insights about how to help organizations provide benefits that keep their employees healthier and more productive.

We invite you to participate in this study. The $75 check enclosed is to thank you for taking time to answer our questions. Participation in this survey is voluntary and will require no more than 15 minutes of your time. The survey should be completed by the human resources or health benefits administrator or the member of the C-suite that is most knowledgeable about health benefits decisions at your company. All responses are private and confidential. Results will be analyzed only in the aggregate and individual responses will not be reported.

Completed surveys can be returned using the provided postage paid envelope or completed online by using the internet address below and entering your unique passcode. Please complete the survey in the next two weeks.

Survey Link: www.stanfordhealthstudy.com

Passcode: <<Respondent Passcode>>

If you have any questions about the research, please contact the survey's Research Director, Sara Singer at 650-498-5354 or via email at sara.singer@stanford.edu.

If you have any questions about the survey administration, please contact the survey administrator, Akilah Evans-Pigford at 484-840-4314 or via email at aevans-pigford@ssrs.com. For participant's rights questions, contact 1-866-680-2906.

Thank you for your consideration!

Sincerely,

Sara J. Singer, MBA, Ph.D.
Stanford Graduate School of Medicine

Jeffrey Pfeffer, Ph.D.
Stanford Graduate School of Business

Sample Consent as follows:

STANFORD BUSINESS GRADUATE SCHOOL OF — Stanford MEDICINE

Employee Healthcare Benefits Survey – Research Consent Form

FOR QUESTIONS ABOUT THE STUDY, CONTACT:

Sara Singer
Medical School Office Building
Stanford University
1265 Welch Road room 328
Stanford, CA 94305
650.498.5354

Jeffrey Pfeffer
Faculty East Building E235
Stanford University
655 Knight Way
Stanford, CA 94305
650.723.2915

DESCRIPTION: You are invited to participate in a research study regarding companies' healthcare benefits. We will be studying the context surrounding and the rationale for the decisions companies make with regard to employee health benefits. The purpose is to gain valuable insights about how to help organizations provide benefits that keep their employees healthier and more productive. You will be asked to participate in a survey, including questions about your organization's healthcare benefits. You can choose to complete the survey via hardcopy or via SSRS' secure website. The survey results will be saved and stored online in an encrypted Stanford Box folder.

RISKS AND BENEFITS: The only potential risk to participants is a breach of confidentiality. To prevent such, all data and documents will be kept in Stanford encrypted and HIPAA compliant files in password protected databases. The benefits which may reasonably be expected to result from this study are none, other than a greater awareness of the decisions and knowledge surrounding employee work environments and health. We cannot and do not guarantee or promise that you will receive any benefits from this study.

TIME INVOLVEMENT: Your participation in this experiment will take approximately 15 minutes.

PAYMENTS/REIMBURSEMENTS: You will receive $75.00 as payment for your participation.

The Robert Wood Johnson Foundation is providing financial support and/or material for this study.

PARTICIPANT'S RIGHTS: If you have read or been read this form and have decided to participate in this project, please understand your participation is voluntary and you have the right to withdraw your consent or discontinue participation at any time without penalty or loss of benefits to which you are otherwise entitled. You have the right to refuse to answer particular questions.

Sample Consent continues:

The results of this research study may be presented at scientific or professional meetings or published in scientific journals. However, your identity and the identity of your company will not be disclosed**.**

Identifiers might be removed from identifiable private information, and, after such removal, the information could be used for future research studies or distributed to another investigator for future research studies without additional informed consent from you.

WITHDRAWAL FROM STUDY: The Protocol Director may also withdraw you from the study without your consent for one or more of the following reasons:

- Failure to follow the instructions of the Protocol Director and study staff.
- The Protocol Director decides that continuing your participation could be harmful to you.
- The study is cancelled.
- Other administrative reasons.
- Unanticipated circumstances.

CONTACT INFORMATION:

Questions, Concerns, or Complaints: If you have any questions, concerns or complaints about this research study, its procedures, risks and benefits, or alternative courses of treatment, you should contact the survey's Research Director, Sara Singer at 650-498-5354 or via email at sara.singer@stanford.edu.

Alternate Contact: If you cannot reach the Research Director, please contact Jeff Pfeffer at pfeff@stanford.edu.

If you have any questions about the survey administration, please contact Akilah Evans-Pigford at 484-840-4314 or via email at aevans-pigford@ssrs.com.

Independent Contact: If you are not satisfied with how this study is being conducted, or if you have any concerns, complaints, or general questions about the research or your rights as a participant, please contact the Stanford Institutional Review Board (IRB) to speak to someone independent of the research team at (650)-723-5244 or toll free at 1-866-680-2906. You can also write to the Stanford IRB, Stanford University, 1705 El Camino Real, Palo Alto, CA 94306.

The extra copy of this consent form is for you to keep.

If you agree to participate in this research, please continue or indicate this to the researcher.

eSupplemental Methodology

eSurvey development

We developed the Employee Healthcare Benefits Survey drawing on insights from a qualitative study of 21 leaders at 20 companies.[1] We also used items exploring companies' engagement with and perceived accountability for employee, environmental, consumer, and community health from the Corporate Culture of Health National Survey in order to assess change in key measures since its administration in 2017 to over 1000 U.S. companies.[2] We adapted selected questions from the previous survey and developed new ones through iterative discussion among the authors and feedback from other health benefits experts and survey design experts at SSRS.[3]

For companies that used health benefits consulting firms, the survey asked what the consultants did for the company (Q6). For companies that used health benefits third-party administrators, we asked about the criteria important for the company's choice of a specific vendor (Q9), rating criteria for choosing a benefits administrator on a scale of 0 for completely unimportant to 5 for extremely important. The survey asked what spending information (Q11) and what user experience information (Q12) was included in the health benefits administrator's annual report on the performance of the company's health plan. Additionally, for all respondents, we asked about the elements tracked by either the company or the health benefits administrator (Q19).

<u>Sample</u>

Our survey sample included in-company human resources administrators identified from a Dun and Bradstreet (D&B) database[4] of companies provided by SSRS. In a pilot survey, we randomly sampled 200 companies generated from the D&B Listing. For the main survey, we randomly sampled 1000 companies, which we supplemented with an additional 118 companies. We combined these to comprise our sample. Excluding non-deliverable surveys and ineligible respondents left 1159 total companies sampled, 185 in the pilot and 974 in the main survey. The sample was drawn to be geographically representative of companies in the U.S. and stratified by number of employees (50-99, 100-499, 500+).

© 2025 Pfeffer J et al. *JAMA Health Forum.*

Survey administration

SSRS administered the pilot survey between May and July 2022, and the main survey between November 2022 and April 2023 using multiple administration modalities. SSRS reached out to each organization via mail three times. It also conducted online and phone verification to identify qualified respondents.

Survey response

Of organizations sampled, 251 (21.7%) responded; we then excluded 30 respondents that self-reported fewer than 50 employees, leaving 221. Respondents did not differ significantly from non-respondents on available characteristics as shown in the table below. Response for some variables reflect a subset of respondents due to the survey's skip logic. Item missingness, unrelated to skip patterns was low, <5% for all items.

eTable 1. Sample Characteristics

		Total Responders (N=221)		Non-Responders* (N=908)		
Characteristic	Level/Measure	Count	Percent	Count	Percent	p-value
Size	50-99	67	30.3%	260	28.6%	0.349 (Fisher's exact test)
	100-499	64	29.0%	231	25.4%	
	500+	90	40.7%	417	45.9%	
Census Division	New England	7	4.0%	56	6.2%	0.054 (Chi Square)
	Middle Atlantic	21	12.0%	149	16.4%	
	East North Central	27	15.4%	148	16.3%	
	West North Central	21	12.0%	65	7.2%	
	South Atlantic	40	22.9%	145	16.0%	
	East South Central	10	5.7%	51	5.6%	
	West South Central	19	10.9%	109	12.0%	
	Mountain	5	2.9%	61	6.7%	
	Pacific	25	14.3%	124	13.7%	
	Missing	46		0		
Industry	Agriculture, Forestry, and Fishing	5	2.3%	8	0.9%	0.260 (Fisher's exact test)
	Construction	12	5.4%	55	6.1%	
	Finance Insurance, and Real Estate	16	7.2%	89	9.8%	
	Manufacturing	38	17.2%	167	18.4%	
	Mining	3	1.4%	10	1.1%	
	Retail Trade	22	10.0%	96	10.6%	
	Services	106	48.0%	364	40.1%	
	Transportation, Communications, Electric, Gas, and Sanitary Services	8	3.6%	55	6.1%	
	Wholesale Trade	11	5.0%	64	7.0%	

*Source: SSRS

<u>*Analysis*</u>

Cleaning and weighting

We compared data on company size, given by the D&B database to size category selected by survey respondents. For those that did not match, we used the size category selected by the respondent. Similarly, we compared data on industry using the US Department of Labor's Occupational Safety and Health Administration's Standard Industrial Classification (SIC) Manual[5] codes listed in the D&B database[4] to industry indicators selected by survey respondents. For the majority of respondents (165 of 221), the SIC code provided by the D&B database and the industry identified by survey respondents matched. For those that did not match,

we utilized SIC code identified in the D&B database. Additionally, we separated healthcare and education services organizations from the other service organizations because of their large number in the dataset.

SSRS performed post-stratification weighting using iterative proportional fitting (or "raking") using SPSSINC RAKE, an SPSS extension module that simultaneously balances the distributions of all variables using the GENLOG procedure. The parameters used for raking were industry, company size, and census region. Weights were trimmed at the 2nd and 98th percentiles to prevent individual cases from having too much influence on the final results. The design effect for this survey was 1.64. The corresponding margins of sampling error (MoSE) is plus or minus 7.9 percentage points.

eMeasures

Several composites measured differences in financial and non-financial concerns:

For companies that used a health benefits consulting firm (Q6), the financial composite is the proportion selected by the respondent of the following three tasks: providing cost estimates, including projections of healthcare costs; making recommendations about health plan design, including copayments, deductibles, etc.; and comparing the businesses' health care costs and service utilization with relevant benchmarks. The corresponding non-financial composite is the proportion selected of the following five tasks: advising on the quality or making recommendations for a health benefits administrator; providing information on the effects of health plan design changes on employee behavior; helping monitor employee physical health trends in aggregate, such as healthy days at work; assessing mental health trends in aggregate; and comparing the business' health benefits with relevant benchmarks.

For companies that used a benefits administrator, respondents were asked to rate the importance (on 0-5 scale) of various criteria in selecting an administrator (Q9). The financial composite is the mean importance score of two criteria: the price of administration and pricing of comparable services. The non-financial member experience composite is the mean importance score across six criteria: telephone availability, ease of use of the administrator's website, member satisfaction, quality ratings, ease of locating in-network providers, and employee health outcomes. The other non-financial composite included the remaining six criteria for choosing a

health benefits administrator: the kind of payment they accept (i.e., flat fee, % of claims, other), their size, their prestige or brand recognition, their market share in locations where the company operates, if they are a licensed carrier, and if the company prioritizes prior relationships of company employees with the health benefits administrator.

For companies that used a health benefits administrator that provided an annual report, the financial composite is the proportion of the following five health care spending items (Q11) that were included in the that report: total health care spending, trend in spending over the past several years, spending on the highest costs cases, where personal identity of the case was not disclosed; spending on patients with specific conditions, such as diabetes, hypertension, etc., and spending based on enrollee demographics such as income, gender, and race/ethnicity. The non-financial composite is the proportion of the following seven user experience items (Q12) included in the report: the amount of time employees spent getting questions answered by health benefits administrators, percent of claims initially or ultimately denied; percent of requests for prior authorization denied; number of grievances or appeals field; how often employees postponed filling a prescription, visiting a doctor or having a medical procedure because of cost; or because of an insurance administrator's actions such as denying authorization; and measures of employee satisfaction with their health benefits or the business' health benefits administrator.

All respondents were queried regarding health benefits information tracked by their company (Q19). The financial composite is the proportion selected of the following five items: the absolute level of spending on health benefits, trends in health benefit costs over time, spending on the highest-cost (deidentified) cases, spending on patients with specific conditions, and spending based on enrollee demographics. The non-financial composite is the proportion selected of the following ten items: whether there was tracking of the physical health status of the business' workforce, such as self-reported health, the incidence of diabetes or other diseases in the business' workforce; mental or emotional well-being of the workforce; the employees' opinions about the business' health benefits or health benefits administrator; amount of time employees spend getting questions answered by the health benefits administrator; percent of claims initially or ultimately denied; percent of requests for prior authorization denied; number of grievances or appeals filed; how often employees delayed

filling a prescription, visiting a doctor, or having a medical procedure because of an insurance administrator's actions or because of cost; and how often medical costs have caused employees to forgo other spending.

All respondents were also asked what businesses did with savings, if any, on health benefits (Q23). The proportion selected of the two options benefiting the employer, use of savings within the company's general fund and adding to shareholder earnings, comprise the financial composite. The proportion selected of the two options benefiting the employee, through reduced employee cost sharing and adding back to employee paychecks, (including any 'other' response indicating employee benefit) comprise the non-financial composite.

Company characteristics that we thought might be associated with employer priorities about employee health benefits included the following: company *size* (number of employees: 50-99, 100-999, 1000-9999, 10,000 or more), *risk* for costs of health care (self-insured or not), use of a *benefits consultant* or not, use of *benefits administrator* or not, frequency with which *employee feedback* regarding their health benefits is requested (0=never, not requested, not sure, or refused; 1=less than every other year; 2=every other year; 3=annually; 4=semi-annually; 5=quarterly or more frequently), whether the company's *mission statement* or business objectives reference improving or maintaining employee health and well-being or not, and whether the company has a *formal plan* for health and well-being across at least two of four domains: employee, environmental, consumer, and community, or not. We also included a composite (0-3 scale) counting actions a company may take to create a *culture of health* including: (1) having programs to promote employee health and wellness, (2) offering monetary rewards to employees for healthy lifestyles, and (3) undertaking initiatives such as housing, financial, or tuition assistance to increase access to services that would indirectly improve employee health and well-being.

What companies say matters: Criteria for choosing a health benefits administrator

Q9 Survey Question. On a scale from 0 to 5, where 0 means completely unimportant and 5 means extremely important, how important are the following criteria in your company's choice of a health benefits administrator? Please record a response for each item below.

The table below includes the survey responses. Weighted and non-weighted items are provided. Since the question was asked on a Likert Scale, we have provided the mean for each item and the composite mean for the financial and non-financial member experience composites.

eTable 2. Q9: Financial and Non-Financial Criteria for Choosing a Health Benefits Administrator, Items and Composite Means

Classification	Composite*/item	sample mean	sample N	weighted mean	weighted N
Financial	Cost composite	4.5	182	4.5	174.3
	Pricing of services for comparable doctors, laboratories, and hospitals	4.5	180	4.5	171.2
	Cost or price of the administration	4.5	182	4.5	174.3
Non-Financial: Member Experience	Member experience composite	4.1	182	4.0	174.3
	Ease of locating in-network doctors, labs, and hospitals	4.6	182	4.6	174.3
	Member satisfaction	4.5	181	4.4	174.1
	Employee health outcomes	4.4	180	4.3	173.5
	Ease of use of the administrator's website	4.0	179	4.0	172.2
	Hours of telephone availability to employees	3.5	176	3.5	166.7
	Quality ratings, such as those on HealthCare.gov	3.4	177	3.4	167.5
Non-Financial: Other	Licensed carrier	4.4	174	4.5	166.8
	Kind of payment (i.e., flat fee, % of claims, other)	3.8	174	3.9	165.7
	Market share in locations where you operate	3.6	168	3.5	156.0
	Prior relationships of company employees with the health benefits administrator	3.2	178	3.3	172.0
	Prestige or brand recognition	3.1	178	3.1	170.8
	Overall size	3.0	175	3.0	166.0
	** Imputing where appropriate*				

© 2025 Pfeffer J et al. *JAMA Health Forum.*

What companies and their advisors measure and track: Information reported

Q6 Survey Question. Which of the following, if any, does the health benefits consulting firm do for you? Please select all that apply.

The table below includes the survey responses. Weighted and non-weighted items are provided. The light green indicates the percent of respondents who selected 0, 1, 2, or 3 of the possible financial actions their consulting firm does for them. The darker green indicates the percent of respondents who replied in the affirmative to each of the possible financial actions. The light blue indicates the percent of respondents who selected 0-5 of the different possible non-financial actions their consulting firm does for them. The darker blue indicates the percent of respondents who replied in the affirmative to each off the possible non-financial actions.

eTable 3. Q6: Financial and Non-Financial Actions by Health Benefits Consulting Firms, Items and Composite Means

Classification	Measure	Level/item	sample %	sample N	weighted %	weighted N
Financial Actions	Count: # items selected	0	0.7%	1	0.1%	0.2
		1	4.8%	7	5.7%	8.2
		2	18.5%	27	26.6%	38.2
		3	76.0%	111	67.5%	97.0
	Item Selected (yes/no)	Provides cost estimates, including projections of healthcare costs	93.8%	137	92.2%	132.4
		Makes recommendations about health plan design, including copayments, deductibles, etc.	91.8%	134	91.2%	130.9
		Compares your health care costs and service utilization with relevant benchmarks	84.2%	123	78.2%	112.3
		average over items	90.0%		87.2%	
Non-Financial Actions	Count: # items selected	0	6.2%	9	8.8%	12.6
		1	14.4%	21	16.7%	24.0
		2	33.6%	49	34.9%	50.0
		3	23.3%	34	21.3%	30.5
		4	6.8%	10	3.8%	5.5
		5	15.8%	23	14.6%	20.9
	Item Selected (yes/no)	Compares your health benefits with relevant benchmarks	84.9%	124	77.6%	111.4
		Advises on the quality or makes recommendations for a health benefits administrator	68.5%	100	67.6%	97.0
		Provides information on the effects of health plan design changes on employee behavior	55.5%	81	48.7%	69.9
		Helps monitor employee physical health trends in aggregate, such as healthy days at work	26.7%	39	25.5%	36.6
		Helps monitor employee mental health trends in aggregate	21.9%	32	18.9%	27.2
		average over items	51.5%		47.7%	

The next two tables indicate the total count of all the Financial and Non-Financial Actions selected and the total number of respondents for the question. This allowed us to identify the average items selected by respondent and the proportion of possible items selected by respondent based on the total possible items.

**Financial Actions**	sample	weighted
sum of items	394	375.5
sum of respondents	146	143.6
average items per resp	2.70	2.62
(average/total items) x100	90.0%	87.2%

**Non-Financial Actions**	sample	weighted
sum of items	376	342.1
sum of respondents	146	143.6
average items per resp	2.58	2.38
(average/total items) x100	51.5%	47.7%

Q11,12 Survey Questions. Which, if any, of the following spending information / user experience information (Q11 / Q12) is included in the report? Please select all that apply.

The table below includes the survey responses. Weighted and non-weighted items are provided. The light green indicates the percent of respondents who selected 0-5 of the possible financial information that is included in the report their health benefits administrator provides. The darker green indicates the percent of respondents who replied in the affirmative to each of the possible financial items. The light blue indicates the percent of respondents who selected 0-7 of the possible non-financial user experience information that is included in the report their health benefits administrator provides. The darker blue indicates the percent of respondents who replied in the affirmative to each off the possible non-financial user experience items.

eTable 4. Q11/12: Financial and Non-Financial Information Included in the Health Benefits Administrator's Annual Report, Items and Composite Means

Classification	Measure	Level/item	sample %	sample N	weighted %	weighted N
Finanicial Information Included in Report (Q11)	Count: # items selected	0	1.6%	2	4.8%	4.7
		1	5.6%	7	12.4%	12.2
		2	7.3%	9	11.2%	11.1
		3	15.3%	19	16.1%	15.9
		4	36.3%	45	30.8%	30.4
		5	33.9%	42	24.8%	24.4
	Item selected (yes/no)	Total health care spending	94.4%	117	88.4%	87.3
		Trend in spending over the past several years	87.9%	109	82.8%	81.7
		Spending on highest-cost cases, where identity of the case is not disclosed	84.7%	105	69.2%	68.3
		Spending on patients with specific conditions, such as patients with diabetes, hypertension, etc.	72.6%	90	57.4%	56.6
		Spending based on enrollee demographics, such as income, gender, and race/ethnicity	41.1%	51	32.2%	31.8
		average over items	76.1%		66.0%	
Non-Financial User Experience Information Included in the Report (Q12)	Count: # items selected	0	36.8%	39	42.1%	35.8
		1	16.0%	17	15.7%	13.3
		2	22.6%	24	21.2%	18.0
		3	14.2%	15	9.5%	8.0
		4	5.7%	6	8.8%	7.4
		5	2.8%	3	2.3%	2.0
		6	0.9%	1	0.2%	0.1
		7	0.9%	1	0.4%	0.3
	Item selected (yes/no)	Percent of claims initially or ultimately denied	44.3%	47	39.7%	33.7
		Percent of requests for prior authorization denied	29.2%	31	25.9%	22.0
		Number of grievances or appeals filed	26.4%	28	23.4%	19.9
		Measures of employee satisfaction with their health benefits or your health benefits administrator	24.5%	26	17.2%	14.6
		Amount of time employees spend getting questions answered by health benefits administrator(s)	12.3%	13	10.5%	8.9
		How often employees postponed filling a prescription, visiting a doctor, or having a medical procedure because of cost	8.5%	9	13.3%	11.3
		How often employees delayed filling a prescription, visiting a doctor, or having a medical procedure because of an insurance administrator's actions, such as denying authorization	7.5%	8	6.4%	5.4
		average over items	21.8%		19.5%	

The next two tables indicate the total count of all the financial and non-financial user experience items selected and the total number of respondents for the question. This allowed us to identify the average items selected by respondent and the proportion of possible items selected by respondent based on the total possible financial and non-financial user experience items.

Financial Items	sample	weighted
sum of items	472	325.7
sum of respondents	124	98.7
average items per resp	3.8	3.30
(average/total items)x100	76.1%	66.0%

Non-Financial User Experience Items	sample	weighted
sum of items	162	115.6
sum of respondents	106	84.9
average items per resp	1.5	1.4
(average/total items)x100	21.8%	19.5%

Q19 Survey Question. Does your company, or your health benefits administrator on behalf of your company, track (in a deidentified way) any of the following: Please select all that apply.

The table below includes the survey responses. Weighted and non-weighted items are provided. The light green indicates the percent of respondents who selected 0-5 of the possible financial items they track. The darker green indicates the percent of respondents who replied in the affirmative to each of the possible financial items. The light blue indicates the percent of respondents who selected 0-10 of the different possible non-

financial items they track. The darker blue indicates the percent of respondents who replied in the affirmative to each off the possible non-financial items.

eTable 5. Q19: Financial and Non-Financial Items Tracked, Items and Composite Means

Classification	Measure	Level/item	sample %	sample N	weighted %	weighted N
Financial Items Tracked	Count: # items selected	0	4.8%	9	6.0%	10.3
		1	10.1%	19	15.3%	26.1
		2	18.6%	35	23.2%	39.5
		3	21.3%	40	20.1%	34.2
		4	25.5%	48	20.4%	34.8
		5	19.7%	37	15.0%	25.6
	Item selected (yes/no)	Trends in health benefit costs	78.7%	148	73.6%	125.4
		Spending on highest-cost cases, where identity of the case is not disclosed	76.6%	144	63.6%	108.5
		Absolute level of spending on health benefits	71.3%	134	70.7%	120.6
		Spending on patients with specific conditions, such as patients with diabetes, hypertension, etc.	58.5%	110	48.2%	82.1
		Spending based on enrollee demographics, such as income, gender, race/ethnicity	26.6%	50	22.4%	38.1
		average over items	62.3%		55.7%	
Non-Financial Items Tracked	Count: # items selected	0	27.7%	52	34.2%	58.3
		1	23.4%	44	24.2%	41.2
		2	20.7%	39	20.3%	34.6
		3	8.5%	16	5.6%	9.6
		4	9.0%	17	6.0%	10.2
		5	5.3%	10	4.6%	7.8
		6	1.6%	3	2.3%	3.9
		7	1.1%	2	0.5%	0.8
		8	0.5%	1	0.8%	1.3
		9	0.5%	1	0.2%	0.4
		10	1.6%	3	1.4%	2.4
	Item selected (yes/no)	Physical health status of your workforce, such as self-reported health, incidence of diabetes or other diseases of your company's workforce	44.1%	83	32.5%	55.4
		Percent of claims initially or ultimately denied	31.4%	59	26.3%	44.8
		Your employees' opinions about your health benefits or health benefits administrator	27.7%	52	26.2%	44.7
		Mental or emotional well-being of your company's workforce	25.5%	48	21.1%	36.0
		Number of grievances or appeals filed	21.8%	41	19.4%	33.0
		Percent of requests for prior authorization denied	19.1%	36	16.2%	27.7
		Amount of time employees spend getting questions answered by health benefits administrator(s)	7.4%	14	8.0%	13.6
		How often employees delayed filling a prescription, visiting a doctor, or having a medical procedure because of an insurance administrator's actions (e.g., denying authorization)	6.9%	13	7.1%	12.0
		How often medical costs have caused employees to forego other spending	5.9%	11	5.6%	9.5
		How often employees postponed filling a prescription, visiting a doctor, or having a medical procedure because of cost	5.3%	10	5.6%	9.6
		average over items	19.5%		16.8%	

The next two tables indicate the total count of all the financial and non-financial items selected and the total number of respondents for the question. This allowed us to identify the average items selected by respondent and the proportion of possible items selected by respondent based on the total possible financial and non-financial items.

Financial Items Tracked	sample	weighted
sum of items	586	474.7
sum of respondents	188	170.4
average items per resp	3.1	2.8
(average/total items)x100	62.3%	55.7%

Non-Financial Items Tracked	sample	weighted
sum of items	367	286.2
sum of respondents	188	170.4
average items per resp	2.0	1.7
(average/total items)x100	19.5%	16.8%

Motivation: What companies do with savings

What does your company do with savings, if any, on health benefits (Q23).

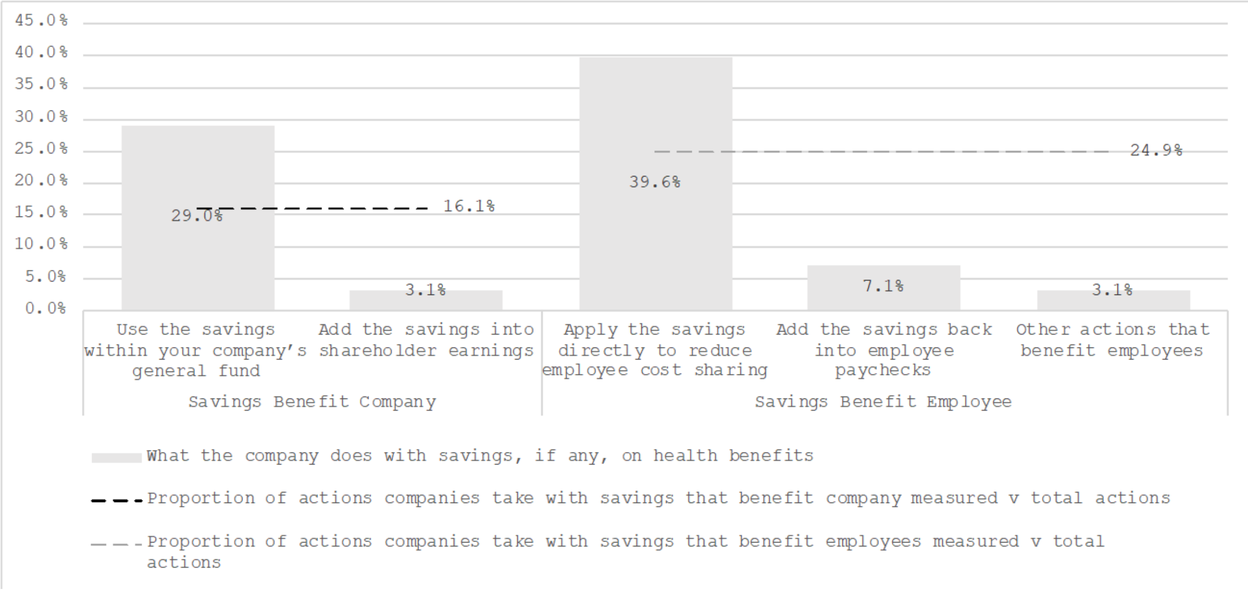

Source: Authors' analysis of the Employee Healthcare Benefits Survey.

Q23 Survey Question. What does your company do with savings, if any, on health benefits?

The table below includes the survey responses. Weighted and non-weighted items are provided. The light green indicates the percent of respondents who selected 0-2 of the possible ways the savings are used to benefit the company. The darker green indicates the percent of respondents who replied in the affirmative to each of the possible items that benefit the employee. The light blue indicates the percent of respondents who selected 0-2 of the different possible ways the savings are used to benefit the employees. The darker blue indicates the percent of respondents who replied in the affirmative to each of the possible items that benefit the employee.

eTable 6. Q23: Uses of Savings that Benefit the Company and Employees, Items and Count Sample Percentages

Classification	Measure	Level/item	sample %	sample N	weighted %	weighted N
Savings Benefit Company	Count: # items selected	0	70.4%	140	68.3%	138.6
		1	28.6%	57	31.2%	63.3
		2	1.0%	2	0.5%	0.9
	Item selected (yes/no)	Use the savings within your company's general fund	26.6%	53	29.0%	58.9
		Add the savings into shareholder earnings	4.0%	8	3.1%	6.3
		average over items	15.3%		16.1%	
Savings Benefit Employee	Count: # items selected	0	48.7%	97	55.8%	113.3
		1	43.7%	87	38.4%	78.0
		2	7.5%	15	5.7%	11.6
	Item selected (yes/no)	Apply the savings directly to reduce employee cost sharing	45.7%	91	39.6%	80.4
		Add the savings back into employee paychecks	9.5%	19	7.1%	14.4
		Other actions that benefit employees	3.5%	7	3.1%	6.4
		average over items	29.4%		24.9%	

The next two tables indicate the total count of all the items selected where savings are used to benefit the company versus the employee and the total number of respondents for the question. This allowed us to identify the average items selected by respondent and the proportion of possible items selected by respondent based on the total possible items that benefit the company and that benefit the employee.

Savings Benefit the Company	sample	weighted
sum of items	61	65.2
sum of respondents	199	202.9
average items per resp	0.3	0.3
(average/total items)x100	15.3%	16.1%

Savings Benefit the Employee	sample	weighted
sum of items	117	101.2
sum of respondents	199	202.9
average items per resp	0.6	0.5
(average/total items)x100	29.4%	24.9%

eReferences

1. Pfeffer J, Vilendrer S, Joseph G, Kim J, Singer SJ. Employers' role in employee health: why they do what they do. *J Occup Environ Med*. 2020;62(11):e601-e610. doi:10.1097/JOM.0000000000001967

2. Kyle MA, Seegars L, Benson JM, Blendon RJ, Huckman RS, Singer SJ. Toward a corporate culture of health: results of a national survey. *Milbank Q*. 2019;97(4):954-977. doi:10.1111/1468-0009.12418

3. About SSRS. SSRS. https://ssrs.com/about/overview/. Accessed July 27, 2023.

4. About us. Dun & Bradstreet. https://www.dnb.com/about-us.html. Accessed July 27, 2023.

5. SIC manual: Occupational Safety and Health Administration. The US Department of Labor. Accessed August 11, 2023. https://www.osha.gov/data/sic-manual
