# Supplementary material for: Emphasis on Financial vs Nonfinancial Criteria in Employer Benefits’ Measurements
Source: JAMA Health Forum. 2025 Jan 31;6(1):e245229. doi: 10.1001/jamahealthforum.2024.5229 (PMC11786228; doi:10.1001/jamahealthforum.2024.5229)
Supplement: Supplement 2. — Data Sharing Statement [file jamahealthforum-e245229-s002.pdf]

# Data Sharing Statement

Pfeffer. Emphasis on Financial vs Nonfinancial Criteria in Employer Benefits' Measurements. *JAMA Health Forum*. Published January 31, 2025. doi:10.1001/jamahealthforum.2024.5229

**Data**
**Data available:** Yes
**Data types:** Deidentified participant data, Data dictionary
**How to access data:** Data will be available upon reasonable request from the corresponding author, Professor Sara Singer at sara.singer@stanford.edu
**When available:** With publication

**Supporting Documents**
**Document types:** Statistical/analytic code, Informed consent form
**How to access documents:** Data will be available upon reasonable request from the corresponding author, Professor Sara Singer at sara.singer@stanford.edu
**When available:** With publication

**Additional Information**
**Who can access the data:** Researchers whose proposed use of the data has been approved.
**Types of analyses:** For any purpose.
**Mechanisms of data availability:** With a signed data access agreement.
